# Surgical excision of heterotopic ossification associated with anti-*N*-methyl-d-aspartate receptor encephalitis: A case report

**DOI:** 10.1016/j.ijscr.2021.106643

**Published:** 2021-12-02

**Authors:** Ken Iida, Yusuke Hashimoto, Shiro Okazaki, Yohei Nishida, Hiroaki Nakamura

**Affiliations:** a Department of Orthopaedic Surgery, Osaka City University Graduate School of Medicine, Osaka, Japan; b Department of Orthopaedic Surgery, Saiseikai Nakatsu Hospital, Osaka, Japan

**Keywords:** Heterotopic ossification, Encephalitis, Surgical excision, Coma, Bilateral knees

## Abstract

**Introduction and importance:**

Heterotopic ossification (HO) associated with Anti-*N*-methyl-d-aspartate (anti-NMDA) receptor encephalitis is rare and the treatment strategy is unclear.

**Case presentation:**

We report the case of a 31-year-old female diagnosed with anti-NMDA receptor encephalitis from Osaka General Medical Center, Osaka, JAPAN that developed heterotopic ossification of the knees during prolonged coma. She was unable to walk because of pain and limited range of motion in both knees. Thirty months after the onset of the coma, surgical excision of the heterotopic bone in both knees was performed. The range of motion of both the knees improved markedly and she had no recurrence of heterotopic ossification on a three-year follow-up. Thus, this case can be used as a guide for surgeons with such patients.

**Conclusions:**

We reported a case of heterotopic bone formation in the periarticular region of both bilateral knees in a patient who suffered a 2-month coma following anti-NMDA receptor encephalitis. Surgical excision of the heterotopic bones significantly improved the passive range of motion in both knees. Three years after the operation, the patient had no complaints about her knees, and there was no recurrence of HO.

## Highlights

• A 31-year-old female diagnosed with anti-NMDA receptor encephalitis that developed heterotopic ossification (HO) of the knees during prolonged coma.
• She was unable to walk because of pain and limited range of motion in both knees.
• Surgical excision of the HO significantly improved the passive range of motion in both knees and she had no recurrence of HO on a three-year follow-up.

## 1 Introduction and importance

Heterotopic ossification (HO) is a pathological process of mature, lamellar bone formation in non-osseous tissues (i.e., muscle and connective tissue) [1]. Heterotopic ossification can be caused by soft tissue injuries (e.g., burn injury), central nervous system (CNS) injuries (e.g., spinal cord injury (SCI), tumor, or encephalitis), arthropathies (e.g., hip arthropathy), vasculopathies, or inheritance [2]. Patients with HO may experience symptoms of pain, inflammation, reduced mobility, loss of normal posture, formation of pressure ulcers, and deep venous thrombosis [3].

Neurogenic HO following traumatic brain injury and SCI has been widely reported; however, HO associated with encephalitis is rare [4]. Anti-*N*-methyl-d-aspartate (anti-NMDA) receptor encephalitis was first described by Dalmau et al. in 2007 as an autoimmune disease in which antibodies to the NMDA receptor are associated with ovarian teratomas in young women [5]. While anti-NMDA receptor encephalitis often presents with severe neurologic symptoms and requires long-term intensive care and mechanical ventilation, patients with this condition are easily susceptible to developing HO [6]. The clinical features usually evolve as follows: psychiatric presentations with seizures, conscious disturbance, dysautonomia, and movement disorder, and recovery after treatment [7]. Although the occurrence of excessive, symptomatic HO around the bilateral knees has been rarely described in the literature [8], we present a case of anti-NMDA receptor encephalitis with HO of the bilateral knees; the heterotopic bones of both knees were surgically excised, which led to improved knee functioning. This work has been reported in accordance with the SCARE criteria [9].

## 2 Case presentation

**Fig. 1:**
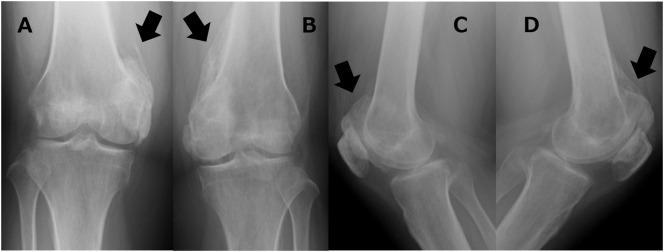
Preoperative anteroposterior and lateral radiographs of the knees showing heterotopic ossification on the peripheral areas (arrow). A: anteroposterior radiographs of the right knees B: anteroposterior radiographs of the left knees C: lateral radiographs of the right knees D: lateral radiographs of the left knees.

**Fig. 2:**
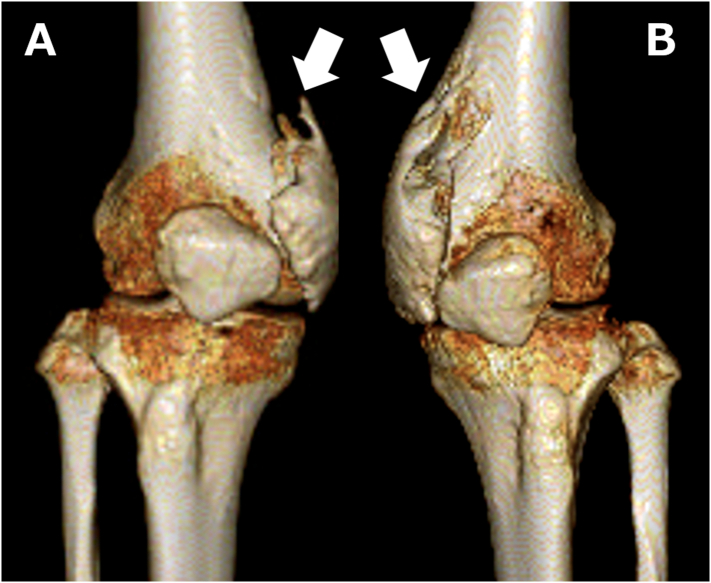
Three-dimensional computed tomography of the knees showing heterotopic ossification on the peripheral areas of knee joints, especially on the medial compartment (arrow). A: Three-dimensional computed tomography of the right knees B: Three-dimensional computed tomography of the left knees.

**Fig. 3:**
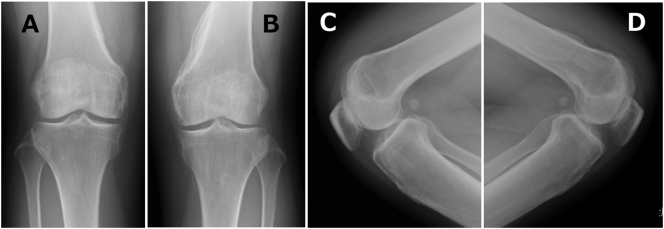
Postoperative anteroposterior and lateral radiographs of both knees showing no recurrence of the ossific mass 36 months post-excision and no change in passive range of motion. A: anteroposterior radiographs of the right knees B: anteroposterior radiographs of the left knees C: lateral radiographs of the right knees D: lateral radiographs of the left knees.

Informed consent has been obtained from the patient, and all identifying details have been omitted. A 31-year-old female suddenly complained of headaches that progressed to a coma. On previous hospital admission (Osaka General Medical Center, Osaka, JAPAN), a physical examination revealed mental confusion, but nervous system examinations were unremarkable. Magnetic resonance imaging (MRI) of the brain was normal, and cerebrospinal fluid (CSF) tests showed that cell counts and glucose (mg/dL) and protein levels (mg/dL) were within normal ranges. However, anti-NMDA receptor antibodies were detected in the CSF, and a pelvic MRI showed edema and slight enhancement of the bilateral ovary. According to the profile, CSF test results, and imaging evidence, the patient was diagnosed with anti-NMDA receptor encephalitis; subsequently, the bilateral ovary was removed, and the patient was treated with antiepileptic therapy over a 60-day coma period. Thereafter, her mental state and speech showed improvement with vigorous and effective treatment, but ambulation did not improve. Eighteen months after the onset of the coma, the patient presented to our hospital on Osaka City University Graduate School of Medicine, Osaka, JAPAN with complaints of limited range of motion and difficulty in walking, and after the patient fell, she was unable to walk because of pain in both her knees. There were no signs of swelling, redness, or heat in either knee joint. The left knee's range of motion was limited to −10° extension and 50° flexion, while the right knee's range of motion was limited to −15° extension and 45° flexion. Anteroposterior and lateral radiographs (Fig. 1) and three-dimensional computed tomography of the knees (Fig. 2) showed HO on the peripheral areas of the knee joints, especially on the medial compartment. All other joints were normal, and the patient's nervous system examinations were unremarkable. Whole-body bone scintigraphy revealed moderate technetium uptake in both knees without strong accumulation in other parts of the body. The patient underwent physical therapy during preoperative rehabilitation two times a week but showed no improvement in her symptoms. Thirty months after the onset of the coma, surgical excision of the heterotopic bone in both knees was performed; bone resection was performed to excise the ossific mass to free the joints. Postoperative radiographs of both knees showed that most of the heterotopic bones had been excised. At the end of the procedure, both knees could achieve a range of motion of 0–135°. Postoperatively, the patient was administered 180 mg of Loxonin® (Loxoprofen; Daiichi Sankyo, Tokyo, Japan) and 1000 mg of etidronate disodium (Didronel; Sumitomo, Osaka, Japan) orally once daily for a total of 12 weeks to inhibit recurrence of the newly excised HO. The range of motion of both the knees improved markedly, reaching 0–130°, and the patient did not experience knee pain while walking. At the final follow-up (36 months postoperatively; 66 months after the coma), the patient did not have pain and could walk independently. A review of the X-ray did not reveal recurrence of HO (Fig. 3).

## 3 Clinical discussion

The incidence of HO has been reported to range from 0.2% to 4% after burn injury, up to 90% after certain types of hip arthroplasty, and 10–53% after central nervous system (CNS) injury [4], [10]. Heterotopic ossification among patients after a burn injury is the percentage of body surface area affected, with burns involving >20% of the body, thereby substantially increasing the likelihood of heterotopic ossification [11]. Heterotopic ossification in patients with a spinal cord injury (SCI) includes the severity and level of the SCI, with injuries to the thoracic and cervical spine resulting in greater severity of HO [12]. In patients after an SCI, HO commonly forms caudad to the level of the injury, and most commonly at the hip. Heterotopic ossification at the peripheral joints is rare [1]. Several studies have reported distinctions between patients who suffered multiple-joint HO following encephalitis [10], [13], [14].

A previous study revealed that the pathogenesis of HO involves three requisite components as follows: osteogenic precursor cells, inducing agents, and a permissive environment [14]. Risk factors for neurogenic HO include the severity of the neurological injury, presence of spasticity compared with flaccid paralysis, and multiple injuries at the time of trauma [15]. Heterotopic ossification is thought to be associated with convulsions and other neurologic factors, as patients with limb convulsions or spasticity have a greater risk of developing HO. Muscle spasticity is common in patients with CNS injuries. Spasticity causes muscle hypoxia and increases the risk of muscle tears due to active or passive mobilization [4].

Patients with anti-NMDA receptor encephalitis usually present with several symptoms, including psychiatric disturbances, memory deficits, seizures, and autonomic abnormalities, according to the time sequence [16]. Prolonged coma and artificial ventilation are important risk factors because limited movements of the extremities are postulated as initiators of neurogenic HO [17]. Furthermore, increased duration of artificial ventilation may alter the patient's body homeostasis in terms of electrolytes and acid-base balance [15]. In our patient, severe neurological symptoms, hypoxia, mechanical ventilation, and delayed rehabilitation were all risk factors for HO.

**Fig. 4:**
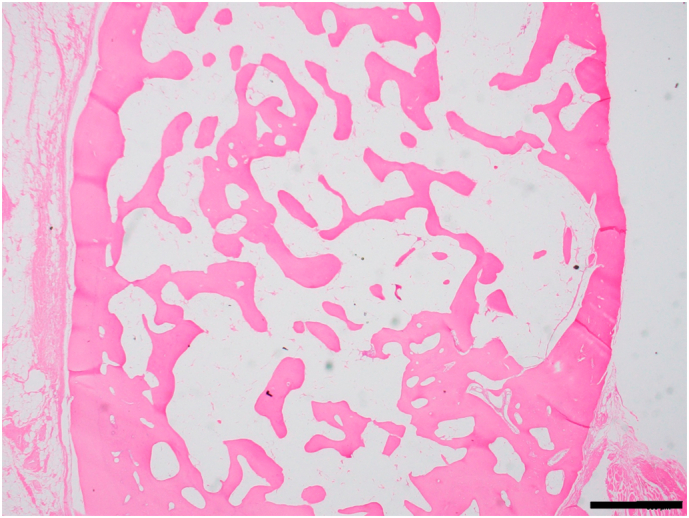
Histologic section of mature lamellar bone with adipose marrow (hematoxylin and eosin stain, scale bars 1 mm).

Generally, HO management includes surgical excisions, pharmacological interventions, and physiotherapy [11]. In our case, physical therapy was exercised under rehabilitation, but the patient did not show significant improvement. Thus, surgical excision of HO around the knee joint was required; surgical excision of HO should be performed when a patient experiences loss of motion or is unable to walk. While the maturity of heterotopic bone is difficult to assess, it is important to avoid the high recurrence rate associated with excision of immature ectopic bone [8]. While most scholars advocate a minimum waiting period of 1 year after heterotopic bone formation prior to surgical excision [1], we performed surgery when the heterotopic bone was found to be fractured 18 months after the coma. Nonetheless, serial roentgenograms indicated that the heterotopic bone was mature, and the level of Alkaline phosphatase (ALP) was normal. Although histology using hematoxylin and eosin stain revealed that the ectopic bone was mature (Fig. 4), slight recurrence occurred; however, etidronate disodium effectively prevented the recurrence of progressive ossification, and a 3-year follow-up confirmed long-term prevention of HO recurrence.

## 4 Conclusion

We reported a case of heterotopic bone formation in the periarticular region of both bilateral knees in a patient who suffered a 2-month coma following anti-NMDA receptor encephalitis. Surgical excision of the heterotopic bones significantly improved the passive range of motion in both knees. Three years after the operation, the patient had no complaints regarding her knees and there was no recurrence of HO.

## Informed consent

The patient provided written informed consent for print and electronic publication of this article.

## Financial disclosure

The authors declared that this study has received no financial support.

## Consent

Written informed consent was obtained from the patient for publication of this case report and accompanying images. A copy of the written consent is available for review by the Editor-in-Chief of this journal on request.

## Research registration

Research Registry was not required.

## Guarantor

Prof. Hiroaki Nakamura.

## CRediT authorship contribution statement

Ken Iida: Conception and design. Drafting of the article.

Yusuke Hashimoto: Conception, and critical revision of the article for important intellectual content.

Yohei Nishida: Interpretation of data.

Shiro Okazaki: Design of the article.

Hiroaki Nakamura: Conception and design, final approval of the article.

## Declaration of competing interest

The authors declare that they have no conflict of interest.
